# Association between low-fat diet and liver cancer risk in 98,455 participants: Results from a prospective study

**DOI:** 10.3389/fnut.2022.1013643

**Published:** 2022-11-18

**Authors:** Linglong Peng, Ling Xiang, Zhiquan Xu, Haitao Gu, Zhiyong Zhu, Yunhao Tang, Yahui Jiang, Hongmei He, Yaxu Wang, Xiaodong Zhao

**Affiliations:** ^1^Department of Gastrointestinal Surgery, The Second Affiliated Hospital of Chongqing Medical University, Chongqing, China; ^2^Department of Clinical Nutrition, The Second Affiliated Hospital of Chongqing Medical University, Chongqing, China; ^3^The Second Affiliated Hospital of Chongqing Medical University, Chongqing, China

**Keywords:** low-fat diet, liver cancer, prevention, prostate, lung, colorectal, ovarian cancer screening trial, cox regression analysis

## Abstract

**Background:**

Low-fat diet reduces the risk of chronic metabolic diseases such as obesity and diabetes, which exhibit overlapping mechanisms with liver cancer. However, the association between low-fat diet and liver cancer risk remains unclear.

**Aim:**

To investigate whether adherence to low-fat diet is associated with a reduced risk of liver cancer in a prospective study.

**Materials and methods:**

Data of participants in this study were collected from the Prostate, Lung, Colorectal, and Ovarian (PLCO) Cancer Screening Trial. A low-fat diet score was calculated to reflect adherence to low-fat dietary pattern, with higher scores indicating greater adherence. Cox regression was used to calculate hazard ratios (HRs) and 95% confidence intervals (CIs) for liver cancer incidence with adjustment for potential covariates. Restricted cubic spline model was used to characterize liver cancer risk across the full range of the low-fat diet score. Prespecified subgroup analyses were used to identify potential impact modifiers. Sensitivity analyses were performed to test the robustness of this association.

**Results:**

A total of 98,455 participants were included in the present analysis. The mean (standard deviation) age, low-fat diet score, and follow-up time were 65.52 (5.73) years, 14.99 (6.27) points, and 8.86 (1.90) years, respectively. During 872639.5 person-years of follow-up, 91 liver cancers occurred, with an overall incidence rate of 0.01 cases per 100 person-years. In the fully adjusted Cox model, the highest versus the lowest quartile of low-fat diet score was found to be associated with a reduced risk of liver cancer (HR_*Q*4 vs. Q1_: 0.458; 95% CI: 0.218, 0.964; *P* = 0.035 for trend), which remained associated through a series of sensitivity analyses. The restricted cubic spline model showed a linear dose–response association between low-fat diet score and liver cancer incidence (*p* = 0.482 for non-linear). Subgroup analyses did not show significant interaction between low-fat diet score and potential impact modifiers in the incidence of liver cancer.

**Conclusion:**

In this study, low-fat diet score is associated with reduced liver cancer risk in the US population, indicating that adherence to low-fat diet may be helpful for liver cancer prevention. Future studies should validate our findings in other populations.

## Introduction

Primary liver cancer is one of the seven most common cancers and the second leading cause of cancer deaths worldwide. In 2020, approximately 905,677 cases were newly diagnosed with liver cancer, and an estimated 830,180 individuals died from liver cancer (1). It is well-established that hepatitis B/C virus infection and alcohol consumption are the main risk factors for liver cancer (2). However, a proportion of cases cannot be explained by traditional risk factors. Emerging evidence concerning diet, including single nutrients and dietary patterns has confirmed a close association between liver cancer risk and diet, and certain dietary patterns are advised for liver cancer prevention (3). For example, in a US population study with average follow-up time of 32 years, the incidence of liver cancer was reduced by a maximum of 39% in participants with a high healthy eating index (4). In a study integrating two Chinese cohorts, a total of 132,837 participants were divided into quartiles based on a vegetable-based dietary pattern, and the risk of liver cancer for participants in the highest quartile was reduced by 42% compared with the lowest quartile (5). Although both dietary patterns were not specifically developed to prevent cancer, they were related to each other and share low-fat dietary components. Low-fat diet is a dietary pattern designed to reduce total fat and calorie intake, which has been shown to be beneficial in reducing the risk of chronic metabolic diseases such as obesity and diabetes (6–8). Additionally, human and animal studies also suggest that low-fat diet has the potential to reduce the secretion of inflammatory cytokines and mediators, including interleukins, tumor necrosis factor-α, Toll-like receptors and complements, and the activity of the transcription factor NF-kB, which was demonstrated to be closely related to increased cancer risk including liver cancer (9–11). However, there are currently large knowledge gaps regarding the association between low-fat diet and liver cancer risk. Thus, the aim of this study was to investigate the association of low-fat diet with the risk of liver cancer in a large population.

## Materials and methods

### Study design and population

All data included in this study were from the Prostate, Lung, Colorectal, and Ovarian (PLCO) Cancer Screening Trial administered by the National Cancer Institute (NCI). The PLCO trial is a large randomized controlled study involving 154,887 participants in ten United States centers from 1993 to 2001, and its main purpose is to determine whether mortality from prostate, lung, colorectal, and ovarian cancers can be reduced using related screening methods in people between the ages of 55 and 74 years (12). According to the design of the PLCO trial, participants were randomly assigned to control or intervention groups in equal proportions after providing informed consent; usual care was received in the control group and screening exams were performed in the intervention group. Several questionnaires, including Baseline Questionnaire (BQ), Diet History Questionnaire (DHQ), and Supplemental Questionnaire (SQX), were completed by the participants in a self-reported manner. The BQ was used to collect the baseline risk factors, such as demographics and medical history, at the time of participant randomization. The DHQ was used to collect the dietary information of participants based on the 137-item Food Frequency Questionnaire (FFQ), and multiple studies have confirmed that the FFQ is a good nutrient assessment pattern (13, 14). The SQX was used to supplement some data not collected by the BQ. Detailed information on the PLCO trial is provided in the literature (12). Our present study was approved by the NCI (Project ID: PLCO-987).

**FIGURE 1:**
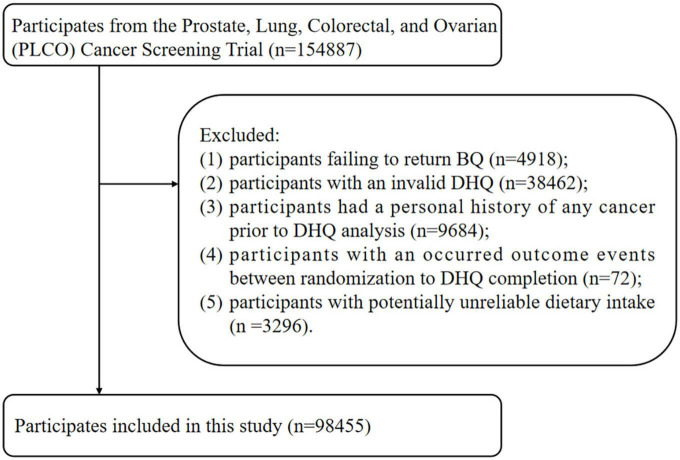
The study flow chart of identifying eligible participants. PLCO, Prostate, Lung, Colorectal, and Ovarian; BQ, baseline questionnaire; DHQ, diet history questionnaire.

Participants for this study were excluded using the following exclusion criteria: (I) participants who did not return the BQ (*n* = 4,918); (II) participants with an invalid DHQ, identified as participants who lacked 8 + frequency responses on the DHQ, whose calorie intake was extreme (the first and last percentile) for each gender as assessed by the DHQ, and the DHQ completion date was available and prior to the date of death (*n* = 38,462); (*n* = 38,462); (III) participants with a personal history of any cancer prior to DHQ analysis (*n* = 9,684); (IV) participants with an outcome event between randomization and DHQ completion, which for the present study were defined as those participants who developed liver cancer, died, or were lost to follow-up (*n* = 72); and (V) participants with potentially unreliable dietary intake, defined as very low or high caloric intake (< 600 or > 3,500 kcal/day for female and < 800 or > 4,200 kcal/day for male) (15) (*n* = 3,296). Finally, a total of 98,455 participants were eligible for inclusion (Figure 1).

### Assessment of low-fat diet score

The low-fat diet score was calculated according to the criteria reported by Shan et al. (15). Specifically, individuals were classified into 11 strata based on each of percentage of energy from carbohydrate, protein, and fat (Supplementary Table 1). For carbohydrate and protein, individuals in the lowest stratum received 0 points and those in the highest stratum received 10 points. For fat, the order of the strata was reversed. Then, the points for the three macronutrients were summed to calculate each participant’s low-fat diet score, which ranged from 0 to 30. Thus, the higher the score, the more closely the participant’s diet followed the pattern of a low-fat diet. In the present study, nutrient variables used for computing the low-fat diet score were extracted from the above-mentioned DHQ. DHQ nutrient variables are calculated from the questionnaire responses by the DietCalc software, which takes into account serving size, food frequency, and other responses, and uses these in conjunction with CSFII nutrient databases to calculate the daily intake of all nutrients (16). Of note, although the dietary information collected through the DHQ was a one-time inquiry of participants’ dietary status over the past 12 months and was not cumulatively updated during follow-up, the reproducibility and validity of the DHQ have been demonstrated elsewhere (17).

### Assessment of covariates

In this study, demographic, lifestyle, and medical information, including sex, race, educational level, arm (intervention or control group), body mass index (BMI), smoking status, pack-years of smoking, history of diabetes, history of liver comorbidities (hepatitis or cirrhosis), and aspirin use, were assessed with the BQ. BMI was calculated as weight (kg) divided by height squared (m^2^). Age at DHQ completion, drinking status, alcohol consumption, and macronutrients intake were assessed with the DHQ. Physical activity level was collected with the SQX and defined as the summarized minutes of self-reported moderate to vigorous activity per week.

### Determination of liver cancer

Individuals diagnosed with primary liver cancer were collected through annual reporting methods including but not limited to self-reports, family reports, and death certificates. Cancer reports were tracked and ascertained by extracting any available medical records. In this study, the end point was the incidence of primary liver cancer which included hepatocellular carcinoma (ICD-O-2, C220) and intrahepatic bile duct cancer (ICD-O-2, C221).

### Statistical analysis

For categorical and continuous covariates with < 5% missing values, modal and median values were used to impute missing data, respectively. The covariate “physical activity level” was imputed by the multiple imputation method as up to 25.3% of the values were missing (18). More detail information of imputation data was shown in Supplementary Table 2, and statistical analyses were performed using the imputed datasets.

**FIGURE 2:**
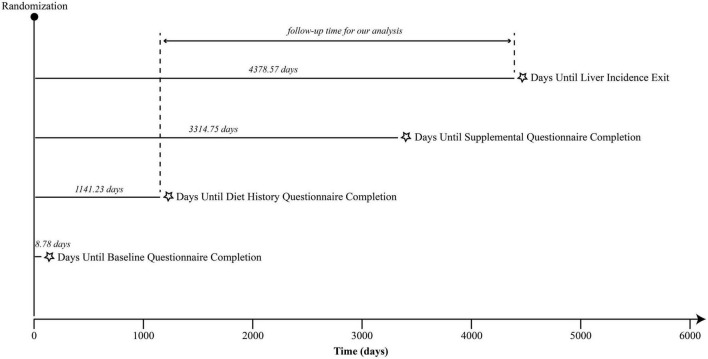
The timeline and follow-up scheme of our study.

A Cox proportional hazards regression model was used to assess the association between low-fat diet and liver cancer risk, and hazard ratios (HRs) and 95% confidence intervals (CIs) were calculated with follow-up time as the time metric. It is worth noting that the follow-up time in this study refers to the date from DHQ completion to the occurrence of liver cancer, death, loss to follow-up, or the end of follow-up (i.e., December 31, 2009), whichever came first (Figure 2). In this model, the low-fat diet scores were divided into quartiles, and the person-years of each quartile were calculated based on the length of the follow-up period. A trend test across quartiles of low-fat diet score for the liver cancer risk estimation was also performed in the Cox regression model by treating the quartiles as a continuous variable with the lowest quartile as the reference group. In multivariate analyses, Model 1 was adjusted for age, sex, and race. Model 2 was further adjusted for education level, arm, BMI, smoking status, smoking pack-years, drinking status, alcohol consumption, aspirin use, history of liver comorbidity, history of diabetes, physical activity level and energy intake from diet. To further characterize liver cancer risk across the full range of the low-fat diet score, a restricted cubic spline model was employed in this study. In addition, we further analyzed the effect of poly-unsaturated fatty acids (PUFA), mono-unsaturated fatty acids (MUFA), and saturated fatty acids (SFA) on the risk of liver cancer using the above-mentioned methods. Specially, PUFA, MUFA, and SFA intakes were derived from DHQ and divided into quartiles, with the lowest quartile as the referent.

A series of subgroup analyses were conducted after stratifying for age (> 65 versus ≤ 65 years), sex (male versus female), BMI (≤ 25 versus > 25 kg/m^2^), smoking status (never versus current/former), drinking status (no versus yes), alcohol consumption (≤ medium versus > medium), history of liver comorbidity (no versus yes), history of diabetes (no versus yes), physical activity (≤ medium versus > medium), and energy intake from diet (≤ medium versus > medium). A *P*_*interaction*_ was computed by comparing models with and without multiplicative interaction terms before performing the above subgroup analyses to avert spurious subgroup effects.

We conducted several sensitivity analyses to test the robustness of our findings. (I) we repeated the primary analysis in participants with non-missing data; (II) we excluded participants with a history of diabetes, as these participants may be prone to a high-fat diet; (III) we excluded participants with a history of liver comorbidity, as these participants may be more likely to develop liver cancer; and (IV) we excluded cases observed within the first 2 and 4 years of follow-up to address the concern of reverse causality.

The statistical analyses were conducted using R 4.1.1 software. A two-tailed *P* value less than 0.05 was considered significant.

## Results

### Baseline characteristics

In the 98,455 included participants, the mean (standard deviation) for low-fat diet score was 14.99 (6.27). Based on the score, we divided participants into quartiles [Quartile 1 (LFD score ≤ 10), *n* = 26,718; Quartile 2 (LFD score 11–15), *n* = 26,149; Quartile 3 (LFD score 16–20), *n* = 24,762; Quartile 4 (LFD score ≥ 21), *n* = 20,826]. The higher the quartile, the more likely the participants were to follow a low-fat dietary pattern. Compared with participants in the lowest quartile group (Quartile 1), participants in the highest quartile group (Quartile 4) were more likely to be older and female and to have a higher educational level, and total carbohydrate and protein intake; but were less likely to have a higher BMI, physical activity level, diet energy and total fat intake. There were more non-smokers and non-drinkers in the highest quartile than in the lowest quartile, and fewer pack-years of current or former smokers and less alcohol consumption by drinkers were observed in the highest quartile. The detailed baseline characteristics of the study population according to quartiles of low-fat diet scores are shown in Table 1.

**TABLE 1:** Baseline characteristics of study population according to overall low-fat diet score.

		Quartiles of overall low-fat diet score				
Characteristics	Overall	Quartile 1 (≤ 10)	Quartile 2 (11–15)	Quartile 3 (16–20)	Quartile 4 (≥ 21)	*P*-value
Number of participants	98,455	26,718	26,149	24,762	20,826	
Low-fat diet score	14.99 ± 6.27	7.33 ± 2.40	12.98 ± 1.42	18.03 ± 1.43	23.75 ± 2.36	0.000
Age	65.52 ± 5.73	65.08 ± 5.63	65.24 ± 5.67	65.73 ± 5.77	66.19 ± 5.79	<0.001
**Sex**						0.000
Male	47216 (47.96%)	14774 (55.30%)	13806 (52.80%)	11265 (45.49%)	7371 (35.39%)	
Female	51239 (52.04%)	11944 (44.70%)	12343 (47.20%)	13497 (54.51%)	13455 (64.61%)	
**Race**						<0.001
White	91218 (92.65%)	25096 (93.93%)	24417 (93.38%)	22401 (90.47%)	19304 (92.69%)	
Non-white	7237 (7.35%)	1622 (6.07%)	1732 (6.62%)	2361 (9.53%)	1522 (7.31%)	
**Education level**						<0.001
College below	62597 (63.58%)	17942 (67.15%)	16899 (64.63%)	15568 (62.87%)	12188 (58.52%)	
College graduate	17352 (17.62%)	4486 (16.79%)	584 (17.53%)	4334 (17.50%)	3948 (18.96%)	
Postgraduate	18506 (18.80%)	4290 (16.06%)	4666 (17.84%)	4860 (19.63%)	4690 (22.52%)	
**Arm**						<0.001
Intervention	50150 (50.94%)	13918 (52.09%)	13441 (51.40%)	12532 (50.61%)	10259 (49.26%)	
Control	48305 (49.06%)	12800 (47.91%)	12708 (48.60%)	12230 (49.39%)	10567 (50.74%)	
Body mass index (kg/m^2^)	27.20 ± 4.79	27.58 ± 4.86	27.47 ± 4.75	27.04 ± 4.72	26.57 ± 4.74	<0.001
**Smoking status**						0.000
Never	47232 (47.97%)	10722 (40.13%)	11889 (45.47%)	12775 (51.59%)	11846 (56.88%)	
Current	8992 (9.13%)	3752 (14.04%)	2557 (9.78%)	1799 (7.27%)	884 (4.24%)	
Former	42231 (42.89%)	12244 (45.83%)	11703 (44.76%)	10188 (41.14%)	8096 (38.87%)	
Smoking pack-years	17.49 ± 26.40	22.72 ± 29.80	18.69 ± 26.92	15.08 ± 24.24	12.14 ± 21.78	0.000
**Drinking status**						<0.001
No	26679 (27.10%)	6023 (22.54%)	6389 (24.43%)	7258 (29.31%)	7009 (33.66%)	
Yes	71776 (72.90%)	20695 (77.46%)	19760 (75.57%)	17504 (70.69%)	13817 (66.34%)	
Alcohol consumption (g/day)	8.78 ± 19.23	14.40 ± 28.94	9.90 ± 18.18	6.05 ± 10.80	3.43 ± 6.36	0.000
**Aspirin use**						0.052
No	52239 (53.06%)	14308 (53.55%)	13781 (52.70%)	13219 (53.38%)	10931 (52.49%)	
Yes	46216 (46.94%)	12410 (46.45%)	12368 (47.30%)	11543 (46.62%)	9895 (47.51%)	
**History of liver comorbidity**						0.978
No	94937 (96.43%)	25759 (96.41%)	25209 (96.41%)	23877 (96.43%)	20092 (96.48%)	
Yes	3518 (3.57%)	959 (3.59%)	940 (3.59%)	885 (3.57%)	734 (3.52%)	
**History of diabetes**						0.116
No	91988 (93.43%)	25032 (93.69%)	24362 (93.17%)	23139 (93.45%)	19455 (93.42%)	
Yes	6467 (6.57%)	1686 (6.31%)	1787 (6.83%)	1623 (6.55%)	1371 (6.58%)	
Physical activity level (min/week)	123.28 ± 108.77	109.20 ± 102.98	119.45 ± 106.92	128.16 ± 109.97	140.34 ± 114.02	<0.001
Energy intake from diet (kcal/day)	1728.69 ± 658.01	1936.74 ± 722.78	1785.82 ± 657.27	1634.16 ± 603.07	1502.44 ± 529.56	0.000
Total Carbohydrate (% energy)	51.99 ± 9.36	43.36 ± 6.86	49.37 ± 6.04	56.42 ± 7.29	61.10 ± 5.89	0.000
Total fat (% energy)	31.78 ± 7.52	39.44 ± 6.15	33.61 ± 4.13	28.47 ± 4.27	23.59 ± 3.97	0.000
Total protein (% energy)	15.44 ± 2.93	14.34 ± 2.72	15.48 ± 2.89	15.28 ± 3.02	16.98 ± 2.39	0.000

Descriptive statistics are presented as (mean ± standard deviation) and number (percentage) for continuous and categorical.

### Association between low-fat diet score and the incidence of liver cancer

During 872639.5 person-years of follow-up, we documented a total of 91 liver cancer cases, with an overall incidence rate of 0.01 cases per 100 person-years. The mean (standard deviation) follow-up length was 8.862 (1.897) years. In the unadjusted model, participants in the highest quartile had a significantly lower risk of liver cancer than those in the lowest quartile (HR_*Q*4 vs. Q1_: 0.369; 95% CI: 0.182, 0.749; *P* = 0.003 for trend) (Table 2). After full adjustment for potential confounders, the inverse association of the low-fat diet score with the risk of liver cancer was also observed (HR_*Q*4 vs. Q1_: 0.458; 95% CI: 0.218, 0.964; *P* = 0.035 for trend) (Table 2). Notably, this inverse relationship was not altered when repeated analysis was performed using non-missing data (HR_*Q*4 vs. Q1_: 0.277; 95% CI: 0.076, 1.010; *P* = 0.032 for trend) (Table 3). For fat components, liver cancer risk was not significantly associated with PUFA (Supplementary Table 3), MUFA (Supplementary Table 4), and SFA (Supplementary Table 5) in the full adjusted model.

**TABLE 2:** Hazard ratios of the association of low-fat diet score with the risk of liver cancer.

Quartiles of low-fat diet score	Number of cases	Person-years	Incidence rate per 100 person-years (95% CI)	HR (95% CI)		
				Unadjusted	Model 1^a^	Model 2^b^
Quartile 1 (≤ 10)	33	232789.8	0.014 (0.010, 0.020)	1.000 (reference)	1.000 (reference)	1.000 (reference)
Quartile 2 (10–15)	28	230061.6	0.012 (0.008, 0.018)	0.856 (0.517, 1.416)	0.869 (0.525, 1.437)	0.898 (0.536, 1.505)
Quartile 3 (16–20)	20	221039.3	0.009 (0.006, 0.014)	0.633 (0.363, 1.103)	0.672 (0.385, 1.174)	0.725 (0.406, 1.295)
Quartile 4 (≥ 21)	10	188748.8	0.005 (0.003, 0.010)	0.369 (0.182, 0.749)	0.428 (0.210, 0.874)	0.458 (0.218, 0.964)
*P*_*trend*_				0.003	0.013	0.035

HR, hazard ratio; CI, confidence interval. ^a^Adjusted for age (years), sex (male, female), and race (white, non-white). ^b^Adjusted for model 1 plus educational level (college below, college graduate, postgraduate), arm (intervention, control), body mass index (kg/m^2^), smoking status (never, current, former), smoking pack-years (continuous), drinking status (no, yes), alcohol consumption (g/day), aspirin use (no, yes), history of liver comorbidity (no, yes), history of diabetes (no, yes), physical activity level (min/week), and energy intake from diet (kcal/day).

**TABLE 3:** Sensitivity analyses on the association of low-fat diet scores with the risk of liver cancer.

Categories	HR _Quartile 4 vs. Quartile 1_ (95% CI)^a^	*P*_trend_
Repeated analysis in participants with non-missing data	0.277 (0.076, 1.010)	0.032
Excluded participants with a history of liver comorbidity^b^	0.383 (0.161, 0.910)	0.025
Excluded participants with a history of diabetes^c^	0.454 (0.194, 1.058)	0.052
Excluded cases observed within the first 2 years of follow-up	0.474 (0.215, 1.042)	0.054
Excluded cases observed within the first 4 years of follow-up	0.425 (0.176, 1.029)	0.051

HR, hazard ratio; CI, confidence interval. ^a^HRs were adjusted for age (years), sex (male, female), race (white, non-white), educational level (college below, college graduate, postgraduate), arm (intervention, control), body mass index (kg/m^2^), smoking status (never, current, former), smoking pack-years (continuous), drinking status (no, yes), alcohol consumption (g/day), aspirin use (no, yes), history of liver comorbidity (no, yes), history of diabetes (no, yes), physical activity level (min/week), and energy intake from diet (kcal/day). ^b^HR was not adjusted for history of liver comorbidity. ^c^HR was not adjusted for history of diabetes.

### Additional analyses

**FIGURE 3:**
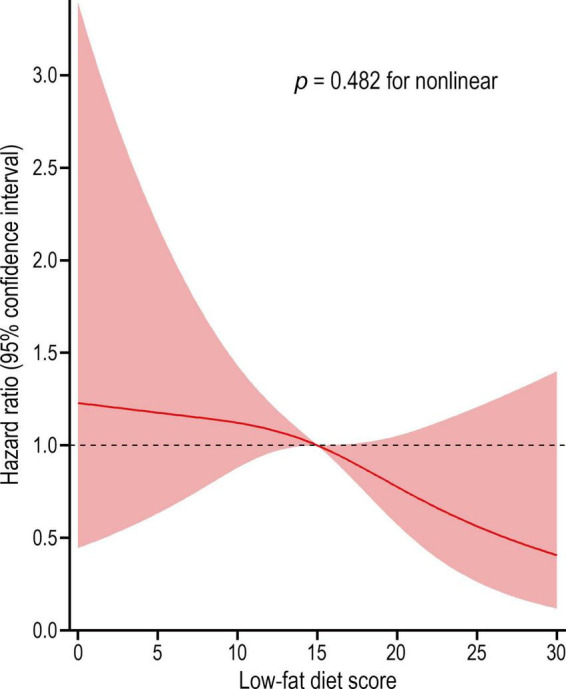
Dose-response association between low-fat diet score and the risk of liver cancer. Hazard ratio was adjusted for age, sex, race, educational level, arm, body mass index, smoking status, smoking pack-years, drinking status, alcohol consumption, aspirin use, history of liver comorbidity, history of diabetes, physical activity level, and energy intake from diet (*p* = 0.482 for non-linear).

We employed a restricted cubic spline model to describe the liver cancer risk across the range of low-fat diet scores, and the results showed that the low-fat diet score was inversely associated with the risk of liver cancer in a linear dose–response manner (*P* = 0.482 for non-linear) (Figure 3). In subgroup analyses, we did not observe a significant interaction between low-fat diet score and age, sex, BMI, smoking status, drinking status, alcohol consumption, history of liver comorbidity, history of diabetes, physical activity level or energy intake from diet in the incidence of liver cancer (all *P* > 0.05 for interaction) (Table 4). In sensitivity analyses, the associations remained similar when we further excluded participants with a history of liver comorbidity or diabetes and excluded cases observed within the first 2 years or 4 years of follow-up, indicating a good robustness of the inverse association of low-fat diet score with liver cancer risk (Table 3).

**TABLE 4:** Subgroup analyses on the association of low-fat diet score with the risk of liver cancer.

Subgroup variable	Number of participates	Number of cases	HR _Quartile 4 vs. Quartile 1_ (95% CI)	*P*_interaction_
**Age (years)**				0.634
≤ 65	24634	22	0.539 (0.184, 1.581)	
> 65	22910	21	0.496 (0.165, 1.496)	
**Sex**				0.344
Male	22145	31	0.638 (0.254, 1.603)	
Female	25399	12	0.325 (0.082, 1.298)	
**Body mass index (kg/m^2^)**				0.796
≤ 25	16695	10	0.394 (0.090, 1.723)	
> 25	30849	33	0.489 (0.198, 1.208)	
**Smoking status**				0.275
Never	22568	11	0.674 (0.188, 2.416)	
Current/Former	24976	32	0.365 (0.132, 1.003)	
**Drinker**				0.887
No	13032	9	0.356 (0.082, 1.547)	
Yes	34512	34	0.553 (0.224, 1.367)	
**Alcohol consumption (g/day)**				0.732
≤ medium	23807	21	0.336 (0.133, 1.004)	
> medium	23737	22	0.633 (0.195, 2.050)	
**History of liver comorbidity**				0.384
No	45851	34	0.398 (0.163, 0.974)	
Yes	1693	9	1.286 (0.317, 5.221)	
**History of diabetes**				0.372
No	44487	30	0.465 (0.194, 1.120)	
Yes	3057	13	0.746 (0.142, 3.928)	
**Physical activity (min/week)**				0.819
≤ medium	24178	14	0.323 (0.072, 1.444)	
> medium	23366	29	0.485 (0.198, 1.188)	
**Energy intake from diet (kcal/day)**				0.339
≤ medium	23772	19	0.631 (0.235, 1.697)	
> medium	23772	24	0.337 (0.075, 1.506)	

HR, hazard ratio; CI, confidence interval.

## Discussion

In this study, we explored whether adherence to low-fat diet is associated with a reduced risk of liver cancer in a large prospective multicenter study. Our results showed a significant inverse association between low-fat diet score and the occurrence of liver cancer, regardless of adjustment for suspected and established confounders. The restricted cubic spline model revealed that this correlation is a non-linear dose-dependent relationship, which means that people who followed a low-fat dietary pattern had a lower risk of liver cancer. In addition, this inverse association remained unchanged even after we excluded several confounding factors through multiple sensitivity analyses.

For decades, the focus on low-fat diet mainly stemmed from the established evidence that low-fat diet can prevent the risk of obesity and diabetes (6, 7). Although dietary recommendations suggest that low-fat diet may be beneficial for cancer prevention, relevant studies are incomplete and controversial due to the wide variety of cancers and conflicting results (19). For example, in a study with a median follow-up time of 8.1 years, the low-fat diet group had a 36% reduced risk in ER-positive and PR-negative breast cancers (20). However, in another cohort study with a mean follow-up of 10 years, the risk of invasive breast cancer was not affected by intervention with low-fat diet in a high-risk population (21). Moreover, the risk for relapse and death was not reduced by the low-fat diet in a population with a very low-risk of breast cancer during a 7.3-year follow-up period (22). In addition to breast cancer, published studies have also linked low-fat diet to pancreatic cancer and skin cancer. In these studies, a reduced risk of pancreatic cancer was observed in women with overweight (BMI ≥ 25 kg/m^2^) among 46,200 participants followed up to 14.7 years (23), but low-fat diet did not decrease the risk of non-melanoma skin cancer (24). One possible reason for these contradictory results is that the definition of low-fat diet was inconsistent among these studies.

To our knowledge, no published data have investigated the association of low-fat diet with liver cancer risk in large populations. The low-fat diet score used in our study has been proven to be very reliable for assessing a low-fat dietary pattern that comprehensively consider the effects of fats, carbohydrates, proteins and energy (15), not just the percentage of fat in total energy (usually < 30% energy) (25). In our study of 98,455 participants with a mean follow-up length of 8.9 years, the incidence of liver cancer was reduced by 55% in participants in the highest quartile of low-fat diet scores compared with the lowest quartile. The risk of liver cancer decreased linearly with increasing low-fat diet score, as an inverse linear association was observed in the restricted cubic spline model (*p* for non-linear = 0.482). For fat components, we did not find a significantly association between liver cancer risk and the intakes of PUFA, MUFA, and SFA in our analyses. However, multiple studies involving fat intake-related dietary patterns, rather than low-fat diet, have obtained contradictory results in relation to liver cancer risk. Polesel et al. reported that the risk of hepatocellular carcinoma can be decreased by 40% in participants with a higher intake of PUFA (26). A significant inverse association between hepatocellular carcinoma risk and total fat intake (HR = 0.80) and MUFA (HR = 0.71) was also observed in a prospective study from Europe (27).

The observed association between low-fat diet and liver cancer risk may be explained by the following mechanisms. Physically, the liver connects to the gut through the bile duct, and the portal vein transports the products of the gut microbiota to the liver (28). Therefore, the crosstalk of gut microbiota between the liver and gut (gut-liver axis) can integrate signals into an interconnected system (29). Dietary patterns alter the gut microbiome balance and subsequently change the immune and inflammatory metabolism landscapes, eventually leading to tumor occurrence and progression (30, 31). For example, it was previously shown that gut microbiota dysbiosis induced by fiber-enriched foods (as inulin-enriched high-fat diet) prone to dysbiosis leads to inflammation, cholestasis, and hepatocellular carcinoma in mice (32). Excessive intake of high-fat diet stimulates the liver to synthesize bile acids, thereby producing large amounts of secondary bile acids in the gut (33), which have been shown to be messengers for microbiota–gut–liver interactions contributing to cancer risk (30, 34), although the underlying mechanisms are unclear. Furthermore, animal and human studies have reported that prolonged consumption of high-fat diet may produce adverse metabolic effects and up-regulate inflammatory mediators, putting the body in a state of chronic inflammation and high postprandial blood glucose and insulin response (11, 35), which is not only involved in the pathogenesis of type 2 diabetes and obesity but also closely related to hepatocarcinogenesis, as liver cancer risk consistently increases with obesity and diabetes (36–38). Conversely, low-fat diet can reduce the secretion of inflammatory mediators and inhibit the activation of tumor-related signaling pathways, ultimately preventing tumor development (39).

This study has significant strengths. This prospective analysis showed for the first time that low-fat diet reduces the incidence of liver cancer in a large population. In addition, good robustness for this inverse association was obtained by multiple sensitivity analyses. For instance, the influence of reverse causation was decreased when excluding cases that occurred in the first 2 years and 4 years of follow-up. In this study, the follow-up time was calculated based on the date of DHQ completion rather than BQ completion, thus ensuring the reliability of low-fat diet score acquisition, and a follow-up time of up to 8 years was sufficient to ensure the occurrence of end-point events. Moreover, the results of this study were extensively adjusted for potential confounders including demographic, lifestyle, medical and dietary factors, thereby minimizing the influence of residual confounders on observed events.

However, several limitations existed in this study. First, we did not find a significant interaction on the incidence of liver cancer between low-fat diet score and potential impact modifiers in a series of subgroup analyses, although the reason may be due to the limited liver cancer cases in each subgroup, resulting in insufficient statistical power for the interaction test. Thus, we cannot provide dietary guidance for specific subgroups based on the results of this study. Second, the low-fat diet score was assessed using a one-time questionnaire without considering that the dietary habits of the participants may change during the follow-up period, which may result in non-differential bias. However, studies have reported that using cumulative averages to assess dietary patterns generally leads to a similar statistical association for disease risk analysis (40), and it is always assumed that the dietary habits of adults generally do not change in nutritional epidemiological studies (41). Third, as the population of our study was American adults aged 55–74 years, we cannot guarantee that the inverse association of low-fat diet with liver cancer risk is applicable for other age groups and non-American populations.

## Conclusion

In conclusion, low-fat diet is associated with a reduced risk of liver cancer in the US population. These findings suggest that adherence to a low-fat diet is helpful for the prevention of liver cancer. Future studies should validate our findings in other populations.

## Data availability statement

The raw data supporting the conclusions of this article will be made available by the authors, without undue reservation.

## Ethics statement

The studies involving human participants were reviewed and approved by the Institutional Review Board of the National Cancer Institute and each screening center. Written informed consent was obtained from all individuals. The patients/participants provided their written informed consent to participate in this study.

## Author contributions

XZ, YW, and LP designed the study. LP, LX, and ZX collected and organized the original data. LP, ZX, and LX analyzed the data. YW, HG, and YT assisted with statistical analysis. LP, ZX, LX, HG, YT, ZZ, YJ, and HH assisted in the interpretation of the results. LP, LX, and XZ drafted the manuscript. All authors contributed to the article and approved the submitted manuscript.
