# Supplementary material for: Association between low-fat diet and liver cancer risk in 98,455 participants: Results from a prospective study
Source: Front Nutr. 2022 Nov 18;9:1013643. doi: 10.3389/fnut.2022.1013643 (PMC9716652; doi:10.3389/fnut.2022.1013643)
Supplement: Supplementary file 1 [file Data_Sheet_1.docx]

SUPPLEMENTARY MATERIALS

**Association Between Low-Fat Diet and Liver Cancer Risk in 98,455 Participants: Results From a Prospective Study**

Linglong Peng^1†^, Ling Xiang^2†^, Zhiquan Xu^1†^, Haitao Gu^1^, Zhiyong Zhu^1^, Yunhao Tang^1^, Yahui Jiang^1^, Hongmei He^1^, Yaxu Wang^1*^, Xiaodong Zhao^3*^

**Supplementary Table 1.** Criteria for determining the low-fat diet score

|  | Low-Fat Diet Score | | |
| --- | --- | --- | --- |
| Points | Total Fat (%energy) | Total Carbohydrate (%energy) | Total Protein (%energy) |
| 0 | ≥41.59 | ≤39.94 | ≤11.76 |
| 1 | 38.47–41.58 | 39.95–43.83 | 11.77–12.91 |
| 2 | 36.29–38.46 | 43.84–46.59 | 12.92–13.71 |
| 3 | 34.41–36.28 | 46.60–48.87 | 13.72–14.38 |
| 4 | 32.72–34.42 | 48.88–50.94 | 14.39–15.01 |
| 5 | 31.02–32.71 | 50.95–52.98 | 15.02–15.63 |
| 6 | 29.23–31.01 | 52.99–55.05 | 15.64–16.29 |
| 7 | 27.26–29.22 | 55.06–57.41 | 16.30–17.01 |
| 8 | 24.92–27.25 | 57.42–60.24 | 17.02–17.92 |
| 9 | 21.71–24.91 | 60.25–64.31 | 17.93–19.25 |
| 10 | ≤21.70 | ≥ 64.32 | ≥19.26 |

| Variable | Before imputation | After imputation | Number (%) with missing data |
| --- | --- | --- | --- |
| Educational level |  |  | 196 (0.20%) |
| College below | 62401 (63.51%) | 62597 (63.58%) |  |
| College graduate | 17352 (17.66%) | 17352 (17.62%) |  |
| Postgraduate | 18506 (18.83%) | 18506 (18.80%) |  |
| Body mass index (kg/m^2^) | 27.21±4.82 | 27.20±4.79 | 1293 (1.31%) |
| Smoking status |  |  | 20 (0.02%) |
| Never | 47212 (47.96%) | 47232 (47.97%) |  |
| Current | 8992 (9.13%) | 8992 (9.13%) |  |
| Former | 42231 (42.90%) | 42231 (42.89%) |  |
| Smoking pack-years | 17.67±26.50 | 17.49±26.40 | 1105 (1.12%) |
| Body mass index (kg/m^2^) | 27.21±4.82 | 27.20±4.79 | 1293 (1.31%) |
| Aspirin use |  |  | 426 (0.43%) |
| No | 51813 (52.85%) | 52239 (53.06%) |  |
| Yes | 46216 (47.15%) | 46216 (46.94%) |  |
| History of liver comorbidity |  |  | 622 (0.63%) |
| No | 94315 (96.40%) | 94937 (96.43%) |  |
| Yes | 3518 (3.60%) | 3518 (3.57%) |  |
| History of diabetes |  |  | 520 (0.53%) |
| No | 91468 (93.40%) | 91988 (93.43%) |  |
| Yes | 6467 (6.60%) | 6467 (6.57%) |  |
| Physical activity (min/week) | 125.15±123.32 | 123.28±108.77 | 24911 (25.30%) |

**Supplementary Table 2.** Distribution of variables with missing data before and after imputation

*Descriptive statistics are presented as (mean ± standard deviation) and number (percentage) for continuous and categorical.*

**Supplementary Table 3*.*** Hazard ratios of the association of poly-unsaturated fatty acids with the risk of liver cancer

| Quartiles of PUFA (g/day) | Number of participants/cases | Person-years | Incidence rate per 100 person-years (95% confidence interval) | Hazard ratio (95% confidence interval) | | |
| --- | --- | --- | --- | --- | --- | --- |
|  |  |  |  | Unadjusted | Model 1^a^ | Model 2^b^ |
| Quartile 1 (≤8.90) | 24629/29 | 219275.4 | 0.013 (0.009, 0.019) | 1.000 (reference) | 1.000 (reference) | 1.000 (reference) |
| Quartile 2 (8.91-12.58) | 24633/24 | 218633.9 | 0.011 (0.007, 0.016) | 0.831 (0.484, 1.427) | 0.749 (0.435, 1.289) | 0.773 (0.438, 1.365) |
| Quartile 3 (12.59-17.61) | 24592/17 | 218193.9 | 0.008 (0.005, 0.012) | 0.590 (0.324, 1.074) | 0.493 (0.270, 0.902) | 0.482 (0.241, 0.963) |
| Quartile 4 (≥17.62) | 24601/21 | 216536.2 | 0.010 (0.006, 0.015) | 0.736 (0.420, 1.291) | 0.561 (0.316, 0.995) | 0.596 (0.247, 1.436) |
| *P*_trend_ |  |  |  | 0.234 | 0.041 | 0.205 |

*PUFA, poly-unsaturated fatty acids; HR, hazard ratio; CI, confidence interval.*

*a Adjusted for age (years), sex (male, female) and race (white, non-white).*

*b Adjusted for model 1 plus educational level (college below, college graduate, postgraduate), arm (intervention, control), body mass index (kg/m^2^), smoking status (never, current, former), smoking pack-years (continuous), drinking status (no, yes), alcohol consumption (g/day), aspirin use (no, yes), history of liver comorbidity (no, yes), history of diabetes (no, yes), physical activity level (min/week) and energy intake from diet (kcal/day).*

**Supplementary Table 4*.*** Hazard ratios of the association of mono-unsaturated fatty acids with the risk of liver cancer

| Quartiles of MUFA (g/day) | Number of participants/cases | Person-years | Incidence rate per 100 person-years (95% confidence interval) | Hazard ratio (95% confidence interval) | | |
| --- | --- | --- | --- | --- | --- | --- |
|  |  |  |  | Unadjusted | Model 1^a^ | Model 2^b^ |
| Quartile 1 (≤14.54) | 24616/20 | 220436.5 | 0.009 (0.006, 0.014) | 1.000 (reference) | 1.000 (reference) | 1.000 (reference) |
| Quartile 2 (14.55-20.92) | 24647/27 | 219173.5 | 0.012 (0.008, 0.018) | 1.361 (0.763, 2.427) | 1.197 (0.668, 2.144) | 1.401 (0.762, 2.577) |
| Quartile 3 (20.93-29.64) | 24591/15 | 217278.8 | 0.007 (0.004, 0.011) | 0.764 (0.391, 1.493) | 0.592 (0.300, 1.169) | 0.845 (0.391, 1.829) |
| Quartile 4 (≥29.65) | 24601/29 | 215750.7 | 0.013 (0.009, 0.019) | 1.491 (0.843, 2.635) | 1.018 (0.561, 1.848) | 2.251 (0.890, 5.688) |
| *P*_trend_ |  |  |  | 0.299 | 0.788 | 0.124 |

*MUFA, mono-unsaturated fatty acids; HR, hazard ratio; CI, confidence interval.*

*a Adjusted for age (years), sex (male, female) and race (white, non-white).*

*b Adjusted for model 1 plus educational level (college below, college graduate, postgraduate), arm (intervention, control), body mass index (kg/m^2^), smoking status (never, current, former), smoking pack-years (continuous), drinking status (no, yes), alcohol consumption (g/day), aspirin use (no, yes), history of liver comorbidity (no, yes), history of diabetes (no, yes), physical activity level (min/week) and energy intake from diet (kcal/day).*

**Supplementary Table 5*.*** Hazard ratios of the association of saturated fatty acids with the risk of liver cancer

| Quartiles of SFA (g/day) | Number of participants/cases | Person-years | Incidence rate per 100 person-years (95% confidence interval) | Hazard ratio (95% confidence interval) | | |
| --- | --- | --- | --- | --- | --- | --- |
|  |  |  |  | Unadjusted | Model 1^a^ | Model 2^b^ |
| Quartile 1 (≤11.99) | 24655/21 | 220800.6 | 0.010 (0.006, 0.015) | 1.000 (reference) | 1.000 (reference) | 1.000 (reference) |
| Quartile 2 (12.00-17.34) | 24593/18 | 218784.1 | 0.008 (0.005, 0.013) | 0.867 (0.462, 1.627) | 0.764 (0.405, 1.441) | 0.905 (0.468, 1.750) |
| Quartile 3 (17.35-25.03) | 24593/25 | 217513.7 | 0.011 (0.008, 0.017) | 1.213 (0.679, 2.167) | 0.966 (0.534, 1.749) | 1.427 (0.717, 2.840) |
| Quartile 4 (≥25.04) | 24614/27 | 215541.0 | 0.013 (0.009, 0.018) | 1.326 (0.749, 2.345) | 0.926 (0.509, 1.683) | 2.082 (0.853, 5.083) |
| *P*_trend_ |  |  |  | 0.192 | 0.948 | 0.056 |

*SFA, saturated fatty acids; HR, hazard ratio; CI, confidence interval.*

*a Adjusted for age (years), sex (male, female) and race (white, non-white).*

*b Adjusted for model 1 plus educational level (college below, college graduate, postgraduate), arm (intervention, control), body mass index (kg/m^2^), smoking status (never, current, former), smoking pack-years (continuous), drinking status (no, yes), alcohol consumption (g/day), aspirin use (no, yes), history of liver comorbidity (no, yes), history of diabetes (no, yes), physical activity level (min/week) and energy intake from diet (kcal/day).*
